# Provision of preventive health care in systemic lupus erythematosus: data from a large observational cohort study

**DOI:** 10.1186/ar3011

**Published:** 2010-05-12

**Authors:** Jinoos Yazdany, Chris Tonner, Laura Trupin, Pantelis Panopalis, Joann Z Gillis, Aimee O Hersh, Laura J Julian, Patricia P Katz, Lindsey A Criswell, Edward H Yelin

**Affiliations:** 1 Division of Rheumatology, University of California, San Francisco, UCSF Box 0920, San Francisco, CA 94143-0920, USA; 2 Division of Rheumatology, McGill University Health Center 1, 650 Cedar Avenue, Room A6-123, Montreal, QC H3G 1A4, Canada; 3 Division of Rheumatology, National Jewish Hospital, 1400 Jackson?Street, Denver, CO 80206, USA; 4 Division of Pediatric Rheumatology, University of California, San Francisco, 533 Parnassus Avenue, San Francisco, CA 94143 - 0107, USA; 5 Division of Rheumatology, University of California, San Francisco, 374 Parnassus Avenue, San Francisco, CA 94143 - 0500, USA

## Abstract

**Introduction:**

Cancer and infections are leading causes of mortality in systemic lupus erythematosus (SLE) after diseases of the circulatory system, and therefore preventing these complications is important. In this study, we examined two categories of preventive services in SLE: cancer surveillance (cervical, breast, and colon) and immunizations (influenza and pneumococcal). We compared the receipt of these services in SLE to the general population, and identified subgroups of patients who were less likely to receive these services.

**Methods:**

We compared preventive services reported by insured women with SLE enrolled in the University of California, San Francisco Lupus Outcomes Study (n = 685) to two representative samples derived from a statewide health interview survey, a general population sample (n = 18,013) and a sample with non-rheumatic chronic conditions (n = 4,515). In addition, using data from the cohort in both men and women (n = 742), we applied multivariate regression analyses to determine whether characteristics of individuals (for example, sociodemographic and disease factors), health systems (for example, number of visits, involvement of generalists or rheumatologists in care, type of health insurance) or neighborhoods (neighborhood poverty) influenced the receipt of services.

**Results:**

The receipt of preventive care in SLE was similar to both comparison samples. For cancer surveillance, 70% of eligible respondents reported receipt of cervical cancer screening and mammography, and 62% reported colon cancer screening. For immunizations, 59% of eligible respondents reported influenza immunization, and 60% reported pneumococcal immunization. In multivariate regression analyses, several factors were associated with a lower likelihood of receiving preventive services, including younger age and lower educational attainment. We did not observe any effects by neighborhood poverty. A higher number of physician visits and involvement of generalist providers in care was associated with a higher likelihood of receiving most services.

**Conclusions:**

Although receipt of cancer screening procedures and immunizations in our cohort was comparable to the general population, we observed significant variability by sociodemographic factors such as age and educational attainment. Further research is needed to identify the physician, patient or health system factors contributing to this observed variation in order to develop effective quality improvement interventions.

## Introduction

Over the last several decades, the prognosis of patients with systemic lupus erythematosus (SLE) has improved dramatically, with five-year survival now above 95% in developed countries [1]. This increased survival has necessitated adjustment of clinical care for this population, with the complications and co-morbidities of the disease gaining greater attention. Infection and malignancy are the two leading causes of mortality in SLE after diseases of the circulatory system [1]. We sought to examine receipt of several preventive services that may influence these outcomes in a large, community-based cohort of individuals with SLE.

Previous literature suggests that individuals with chronic diseases may receive fewer preventive services than the general population [2-5]. In the rheumatic diseases, studies examining cancer screening in rheumatoid arthritis and SLE have yielded similar results [2,5]. The reasons for these findings remain unclear, but hypotheses include that management of the primary disease may dominate provider and patient time, responsibility for preventive services may span several physicians and specialties, and resources may be limited - both in terms of patient time and because chronically ill patients are often underinsured [6]. In SLE, factors specific to the condition, such as the unpredictable nature of disease exacerbations and concerns about vaccine safety in immunocompromised hosts, may also potentially hinder attention to preventive services.

In this study, we sought to examine two categories of preventive services: cancer surveillance (cervical, breast, and colon) and immunizations (influenza and pneumococcal). We compared the receipt of preventive services in SLE to the general population, and identified specific subgroups of patients with SLE who are less likely to receive these services. In addressing this latter aim, we attempted to examine not only characteristics of individuals (for example, sociodemographic and disease related factors), but also characteristics of health systems (for example, number of outpatient visits, involvement of generalist providers or rheumatologists in patient care, type of health insurance) and characteristics of neighborhoods (that is, neighborhood poverty level).

## Materials and methods

### Data sources

#### LOS

Data were derived from the University of California, San Francisco (UCSF) Lupus Outcomes Study (LOS), a longitudinal observational study of 1,179 English-speaking individuals with SLE. Details on study methodology have been reported previously [7]. Briefly, study respondents participate in an annual telephone survey, providing information about their demographic characteristics, socioeconomic status, medications, disability, general health and social functioning, health care utilization, health insurance coverage, and disease activity [7]. Recruitment for the study took place in several settings in an attempt to capture the full spectrum of SLE, including academic rheumatology offices (23%), community rheumatology offices (11%), and community-based sources such as SLE support groups, the Internet, and media advertisements (66%). All patients have carried a diagnosis of SLE from a physician, and we further confirmed these diagnoses by a formal review of the medical record to document American College of Rheumatology Criteria for SLE [8].

Items regarding receipt of preventive health services were introduced in the fourth annual LOS interview, conducted between March 2005 and February 2006; 797 individuals participated in that interview. Of the 797, 14 (2%) were excluded from the sample. Four individuals were excluded because they resided outside the United States at the time of the interview. Ten individuals were excluded because they reported having no health insurance; this group was too small to make robust conclusions regarding lack of insurance. Of the remaining 783 individuals, 5% were dropped from the sample because of the following missing data: below poverty status (n = 10; 1%); HMO (n = 18, 2% did not know if they were in a HMO or not); area poverty level (n = 13; 2% lived in areas with no geocoded data either because they resided in new residential areas that were developed after the 2000 Census was conducted, or because they had moved since the geographic match was performed). For the analysis comparing LOS service receipt to population controls (described below), we included 685 of these respondents; men were excluded (n = 57) to facilitate comparisons with the controls. For the second part of the study examining predictors of receipt of preventive services, men were included, yielding a total sample of 742 individuals.

#### CHIS

For the population comparison, we analyzed primary data from the California Health Interview Survey (CHIS), a random-digit-dial telephone survey administered to households in California [9]. The CHIS sample is representative of California's non-institutionalized population. We chose CHIS because the survey included all the variables of interest, and because a majority of LOS respondents reside in California. Response rates of CHIS are comparable to other statewide telephone surveys (approximately 49.8% among adults interviewed in 2005). We report receipt of preventive services in the LOS compared to two subsamples of CHIS. The first included the overall CHIS sample as a general population comparison, and the second included only CHIS respondents reporting chronic medical conditions (asthma, diabetes mellitus, or heart disease). Among the 4,515 individuals with chronic diseases, 1,595 (35%) had asthma, 3,399 (75%) had diabetes, and 3,255 (72%) had heart disease.

For most preventive services, we analyzed CHIS interviews from the same calendar year as these services were queried from LOS respondents (2005). However, for pneumococcal immunization, we used CHIS data from 2003, the most recent year in which data on this service were available. To more closely approximate the LOS population, we included only data from CHIS respondents ≥ 18 years, who spoke English, were female and reported having health insurance. This resulted in a sample size of 18,013 for the CHIS general population comparison, and 4,515 for the CHIS chronic medical condition comparison.

### Measures

We examined receipt of five preventive care services, including cancer screenings (mammography, colon cancer screening, and cervical cancer screening), and immunizations (influenza and pneumococcal). For cancer screening, we evaluated receipt of care advocated by the United States Preventive Services Task Force recommendations [10]. We examined screening mammography over the previous year in women ≥ 40 years with no history of breast cancer. Cervical cancer screening was examined in the previous year among women 18 to 65 years who did not report a hysterectomy and had no history of cervical cancer. Colon cancer screening (defined as a colonoscopy in the last 10 years or a flexible sigmoidoscopy plus fecal occult blood in the last five years) was examined in those ≥ 50 years with no history of colorectal cancer.

We used the Centers for Disease Control (CDC) recommendations and the recently developed quality indicators for SLE to determine eligibility for immunizations [11-13]. We examined influenza immunization in the previous year among individuals receiving immunosuppressive therapy or age ≥ 50 years. For pneumococcal immunization, we determined whether individuals receiving immunosuppressive therapy or those ≥ 65 years had ever received the vaccine.

We applied the same inclusion and exclusion criteria to the CHIS data. For immunizations, eligibility for the CHIS general population was defined by age (≥ 50 years for influenza, and ≥ 65 years for pneumococcal). For the CHIS population reporting chronic medical conditions, all respondents were considered eligible for influenza immunization, and those ≥ 50 years were considered eligible for pneumococcal vaccine (the CHIS survey only queries receipt of pneumococcal vaccine in those ≥ 50 years).

We considered factors previously associated with receipt of preventive services as potential covariates when examining predictors of receipt of preventive services in the LOS. These included sociodemographic factors such as age, sex, race/ethnicity (Caucasian versus other), education (high school education or less, some college, or college graduate), and poverty (household income less than 125% of the Federal poverty threshold). To examine the influence of disease status, we used the Systemic Lupus Activity Questionnaire (SLAQ), a self-report instrument [14,15], and the Short Form-36 Physical Functioning Scale (SF-36) [16].

Because we were interested in whether health care access variables influence the receipt of preventive care, we also considered: 1) the total number of physician visits over the last year; 2) whether a generalist provider was involved in care (including all physicians or nurse practitioners functioning as primary care providers); 3) whether a rheumatologist was involved in care; 4) the type of health insurance used (public health insurance, including Medicare or Medicaid, or private health insurance, including employer-based plans). The health insurance variables were further stratified by enrollment in a health maintenance organization (HMO), given that some HMOs, particularly prepaid group practices, place special emphasis on preventive care [17,18].

Previous research suggests that community characteristics may hinder access to preventive care in some populations [19]. We therefore evaluated whether a contextual variable, neighborhood poverty, influenced receipt of preventive care services. To obtain information about study participants' neighborhood poverty, data from the 2000 U.S. Census were matched to participants' residential addresses through a process known as geocoding, described in detail elsewhere [20]. Geocoding procedures were conducted using the Environmental Systems Research Institute ArcGIS software by Sonoma Technology (Petaluma, CA, USA).

All respondents provided informed consent to participate in the study. The UCSF Committee on Human Research approved the study protocol.

### Statistical analysis

We compared unadjusted frequencies of receiving preventive services in the LOS to the two CHIS samples. We used SUDAAN 10.0 (Research Triangle Park, NC, USA) in order to account for the CHIS sampling design. Because the samples differed significantly in terms of age and education, we calculated age and education standardized estimates from CHIS to reduce confounding.

We used multivariate logistic regression models to examine whether specific subgroups of patients in the LOS were less likely to receive each preventive service. Each of the models included demographic factors, health care access factors, health status, and neighborhood poverty level. We used SAS 9.2 (SAS Institute, Cary, NC, USA) for this portion of the analysis.

In addition to examining each preventive service individually, we were also interested in examining the overall quality of preventive care in the LOS. We therefore developed pass frequencies [21] (defined as the percentage of times that each service was received among eligible individuals) for the two immunizations, three cancer screening procedures, and all five of these services combined. In these analyses, respondents contributed one observation for each eligible service, and thus there were between one and five observations per respondent. We accounted for these repeated measures in our regression models. We present the adjusted pass frequencies from these models, which provide estimates of the probability of individuals receiving preventive services controlling for all of the other variables of interest. This analysis was completed in SUDAAN 10.0.

## Results

### Patient characteristics

The characteristics of subjects enrolled in the LOS and CHIS are listed in Table 1. To facilitate comparisons with CHIS, males were excluded from the comparative analysis between the two samples (the LOS analysis sample consists of 685 women and 57 men). Both samples are primarily Caucasian (among individuals included in the LOS analysis, 68% were Caucasian, 9% Latino, 7% African-American, 8% Asian/Pacific Islander, and 6% other; among individuals in CHIS, 75% were Caucasian, 9% Latino, 5% African-American, 7% Asian/Pacific Islander, and 4% other). The number of individuals living below poverty was similar between the samples. Although levels of college education were comparable between the two samples, LOS respondents reported higher educational attainment overall. A similar percentage of individuals in the two samples were enrolled in Medicaid. However, a higher percentage of LOS respondents report Medicare coverage. In the LOS, 19% of individuals reported having no primary care provider, and 16% reported that a rheumatologist was not involved in care. Respondents had a mean of 16 physician visits per year.

**Table 1:** Characteristics of women in the Lupus Outcomes Study (LOS) and the California Health Interview Survey (CHIS)

Characteristic	LOS N = 685	CHIS N = 18,013	CHIS, with chronic conditions* N = 4,515
***Sociodemographics***			
Age, *mean (SD)*	50.1 (12)	44.0 (16)	48.7 (28)
Race/Ethnicity, *n (%)*			
Caucasian	468 (68)	13,544 (63)	3,464 (66)
Non-caucasian	217 (32)	4,469 (37)	1,051 (34)
Education, *n (%)*			
<High school or High school graduate	95 (14)	4,810 (32)	1,384 (36)
Some college	322 (47)	5,522 (29)	1,535 (32)
College	268 (39)	7,681 (39)	1,596 (33)
Below Poverty, *n (%)*			
No	602 (88)	4,068 (90)	16,812 (92)
Yes	83 (12)	447 (10)	1,201(8)
***Health Care Access***			
Insurance, *n (%)*			
Medicaid	33 (6)	849 (5)	338 (7)
Medicare	260 (38)	2,012 (7)	774 (13)
Private/Employer	392 (57)	15,152 (88)	3,403 (81)
Insurance category, *n (%)*			
HMO and public insurance (Medicare or Medicaid)	61 (9)	230 (2)	68 (2)
HMO and private insurance	153 (22)	7,967 (52)	1,743 (47)
No HMO and public insurance (Medicare or Medicaid)	235 (34)	2,631 (10)	1,044 (17)
No HMO and private	242 (35)	7,185 (37)	1,660 (34)
Physicians			
Generalist involved in health care, *n (%)*	557 (81)		
Rheumatologist involved in health care, *n (%)*	573 (84)		
Total number of physician visits in one year, *mean (SD)*	16.0 (10.4)		
***Health status***			
SLAQ score, *mean (SD)*	13.0 (8)		
SF-36 Physical Function, *mean (SD)*	57.7 (29)		
***Contextual Factor***			
Neighborhood Poverty, *n (%)*			
No	627 (92)		
Yes	58 (8)		

*Chronic conditions examined in CHIS include self-reported diabetes mellitus, asthma or heart disease.

Abbreviations: LOS, Lupus Outcomes Study; CHIS, California Health Interview Survey; HS, high school; SLAQ, Systemic Lupus Activity Questionnaire; SF-36, Short-Form 36 Health Survey; HMO, health maintenance organization.

### Receipt of preventive health services in the LOS versus CHIS

Table 2 summarizes the percentage of eligible patients enrolled in the LOS and CHIS who received recommended cancer screening and immunizations. We found that the crude frequency of receipt of services in persons with SLE was comparable to the general population for almost all services examined, with the exception of receipt of pneumococcal immunization. LOS respondents were less likely to report pneumococcal immunization than the CHIS general population, but frequencies were similar to those with chronic conditions enrolled in CHIS. Those enrolled in CHIS were less likely to receive influenza immunization than LOS respondents. In addition to examining the crude frequencies reported in Table 2, we also standardized the LOS sample to CHIS by age and education. These standardized frequencies yielded the same conclusions as those discussed above.

**Table 2:** Receipt of preventive services by women in the Lupus Outcomes Study and the general population

Service (LOS eligible group)	LOS 2005		**CHIS 2005**^§^		**CHIS 2005 **^§^**, with chronic conditions**	
	N (%)	95% CI	N (%)	95% CI	N (%)	95% CI
Cervical cancer screening^1 ^(women ≥ 65 years with a uterus)*	302 (70)	66 to 74	9,283 (73)	72 to74	1,917(74)	71 to 76
Mammogram^2 ^(≥ 40 years)*	384 (70)	67 to 74	8,875 (68)	67 to 69	2,449 (69)	67 to 71
Colon Cancer^3 ^(≥ 50 years)*	221(62)	57 to 67	5,355 (57)	55 to 58	1,688 (59)	56 to 61
Influenza vaccine^4 ^(≥ 50 years or immunosuppressed)^†^	338 (59)	55 to 63	4,010 (42)	40 to 43	2,016 (38)	36 to 40
Pneumococcal vaccine^5 ^(≥ 65 years or immunosuppressed)^†^	272 (60)	56 to 65	3,074 (70)	68 to 72	1,756 (57)	54 to 60

§All data are from CHIS 2005, except for pneumococcal vaccine, for which the most recent CHIS data are from 2003. Chronic conditions examined in CHIS include self-reported diabetes mellitus, asthma or heart disease.

1. Compared to CHIS respondents 18 to 65 years with a uterus and no history of cervical cancer with health insurance.

2. Compared to CHIS ≥ 40 years, with no history of breast cancer with health insurance.

3. Compared to CHIS ≥ 50 years, with no history of colon cancer with health insurance.

4. Compared to the CHIS respondents ≥ 50 years with health insurance. For the CHIS sample with chronic conditions, all ages were considered eligible for the vaccine.

5. Compared to the CHIS general population age ≥ 65 with health insurance. For the CHIS sample with chronic conditions, immunization information was available for respondents ≥ 50 years.

*United States Preventive Services Task Force guidelines.

†Centers for Disease Control and Prevention guidelines.

Abbreviations: LOS, Lupus Outcomes Study; CHIS, California Health Interview Survey.

### Predictors of receipt of individual preventive health services in the LOS

Consistent with previous literature on the receipt of preventive health services in the general medical population, different predictors were identified for the different services examined (Table 3). Older individuals were more likely to receive four of the services: influenza immunization, pneumococcal immunization, colon cancer screening and mammography. Race/ethnicity did not predict the receipt of most services, except that non-Caucasian LOS respondents were more likely to report mammography. Those with a college education were more likely to receive most services, although this only reached statistical significance for cervical cancer screening and immunizations. Individuals living below poverty were less likely to receive cervical cancer screening. Although enrollment in an HMO did influence receipt of preventive services, the findings varied across services. For example, those enrolled in HMOs appeared more likely to receive immunizations, but less likely to receive cancer screening.

**Table 3:** Predictors of receiving preventive services in men and women enrolled in the Lupus Outcomes Study

	Immunizations		Cancer Screening		
		
	Influenza Vaccine (n = 626)	Pneumococc al Vaccine (n = 491)	Colon Cancer Screening (n = 389)	Mammogram (n = 545)	Cervical cancer Screenin g (n = 430)
	Odds Ratio (95% CI)				
***Sociodemographics***					
Age (per 10 units)	**1.3 (1.1, 1.5)**	**1.4 (1.2, 1.7)**	**1.7 (1.2, 2.5)**	**1.4 (1.1, 1.8)**	1.2 (1.0, 1.5)
Female	1.1 (0.6, 2.1)	1.1 (0.5, 2.2)	0.5 (0.2, 1.2)	**--**	--
Non-White	0.7 (0.5, 1.1)	0.7 (0.4, 1.1)	0.8 (0.5, 1.4)	**1.6 (1.0, 2.6)**	1.2 (0.7, 2.0)
Education					
High School Graduate	0.7 (0.4, 1.3)	0.9 (0.4, 1.7)	0.9 (0.4, 1.9)	1.3 (0.7, 2.6)	1.5 (0.7, 3.4)
Some College	**0.5 (0.3, 0.8)**	**0.5 (0.2, 0.9)**	0.8 (0.4, 1.5)	0.8 (0.4, 1.4)	**0.4 (0.2, 0.9)**
College Graduate (referent)					
Income					
Below Poverty	0.7 (0.5, 1.1)	0.8 (0.5, 1.2)	0.9 (0.5, 1.5)	1.0 (0.6, 1.5)	**0.4 (0.2,0.6)**
***Health Care Access***					
Insurance					
HMO and Public Insurance (Medicaid, Medicare)	**2.5 (1.2, 5.3)**	**4.5 (1.8, 11.3)**	**0.4 (0.2, 1.0)**	0.7 (0.3, 1.4)	0.4 (0.2, 1.1)
HMO and Private	0.9 (0.6, 1.5)	**2.0 (1.1, 3.4)**	**0.5 (0.2, 0.8)**	0.8 (0.5, 1.4)	**0.4 (0.2, 0.6)**
Insurance					
No HMO, Public	0.9 (0.5 to 1.4)	1.5 (0.9 to 2.6)	0.7 (0.3, 1.2)	0.6 (0.4, 1.1)	**0.4 (0.2, 0.8)**
Insurance (Medicaid, Medicare)					
No HMO, Private Insurance (referent)					
Physician Visits					
Generalist involved in health care	**1.6 (1.0, 2.5)**	**1.8 (1.0, 3.0)**	0.6 (0.3, 1.1)	1.5 (0.9, 2.5)	**2.2 (1.3, 3.7)**
Rheumatologist involved in health care	**2.3 (1.4, 3.8)**	1.5 (0.8, 2.9)	0.8 (0.4, 1.5)	1.4 (0.8, 2.3)	1.0 (0.5, 2.0)
Total physician visits (per five visits)	1.1 (1.0, 1.2)	1.1 (1.0, 1.3)	**1.3 (1.1, 1.5)**	**1.2 (1.0, 1.3)**	**1.2 (1.0. 1.4)**
***Health Status***					
SLAQ Score (per 10 units)	**0.7 (0.5, 1.0)**	0.7 (0.5, 1.0)	1.2 (0.8, 1.8)	1.0 (0.8, 1.4)	1.2 (0.8, 1.7)
SF-36 Physical Function (per 10 units)	**0.9 (0.8, 1.0)**	**0.9 (0.8, 1.0)**	1.0 (0.9, 1.1)	**1.2 (1.1, 1.3)**	**1.2 (1.1, 1.3)**
***Contextual Factor***					
Neighborhood Poverty Area	1.4 (0.7, 2.5)	1.2 (0.6, 2.4)	2.2 (0.9, 5.4)	0.9 (0.4, 1.7)	1.6 (0.6, 4.1)

Abbreviations: SLAQ, Systemic Lupus Activity Questionnaire; SF-36, Short-Form 36 Health Survey; HMO, Health Maintenance Organization. Bolded values indicate *P *< 0.05.

Those reporting a visit to a generalist physician in the last year were more likely to receive many of the preventive services examined (influenza and pneumococcal immunizations, cervical cancer screening). Individuals seeing a rheumatologist in the last year were more likely to receive influenza immunizations. Total physician visits increased the likelihood of receiving services, reaching statistical significance for all cancer screening tests. More disease activity, as measured by the SLAQ, was associated with fewer immunizations, as was higher physical functioning. We did not see any significant effects by neighborhood poverty level once personal socioeconomic status was taken into account.

### Predictors of receipt of overall preventive health services in the LOS

Table 4 lists the pass frequencies for immunizations, cancer screening tests, and all preventive services combined in the LOS. The first sample of 1,117 observations represents patients who were eligible for either influenza or pneumococcal immunizations. Since one person may be eligible for both, this sample includes repeated measures. The sample in the second column represents 1,364 observations eligible for colon cancer screening, mammography or cervical cancer screening. The last sample of 2,481 observations combines the previous two samples to represent all observations eligible for preventive services.

**Table 4:** Adjusted pass frequencies for receipt of preventive services by men and women in the Lupus Outcomes Study

	Immunizations (n = 1117)	Cancer Screening (n = 1364)	All Services (n = 2481)
***Sociodemographics***	Percent (95% CI)		
Age			
18 to 34	**0.42 (0.32,0.52)**	**0.63 (0.52,0.74)**	**0.48 (0.40,0.56)**
35 to 54	**0.58 (0.54,0.63)**	**0.65 (0.61, 0.70)**	**0.62 (0.59, 0.66)**
55 to 64	**0.59 (0.52,0.65)**	0.70 (0.66,0.75)	**0.66 (0.62,0.70)**
65+ (*referent*)	0.72 (0.64,0.81)	0.76 (0.70,0.83)	0.75 (0.69,0.80)
Gender			
Female	0.57 (0.47, 0.67)	0.73 (0.59, 0.88)	0.60 (0.51, 0.69)
Male (*referent*)	0.59 (0.56, 0.63)	0.68 (0.65, 0.71)	0.64 (0.62, 0.67)
Education			
HS Grad or less	**0.47 (0.38,0.57)**	**0.63 (0.56,0.71)**	**0.57 (0.51,0.64)**
Some College	**0.58 (0.53,0.62)**	**0.66 (0.61,0.70)**	**0.62 (0.59,0.65)**
College (*referent*)	0.64 (0.59,0.69)	0.73 (0.69,0.77)	0.69 (0.65,0.73)
Race/Ethnicity			
White	0.61 (0.57, 0.65)	0.67 (0.64, 0.71)	0.65 (0.62, 0.68)
Non-White (*referent*)	0.54 (0.48, 0.60)	0.70 (0.65, 0.75)	0.62 (0.59, 0.66)
Income			
Above Poverty	0.60 (0.56,0.63)	0.68 (0.65,0.71)	0.64 (0.62,0.67)
Below Poverty (*referent*)	0.54 (0.44, 0.64)	0.72 (0.64, 0.79)	0.64 (0.57, 0.71)
***Health Care Access***			
Insurance			
HMO with Public Insurance (Medicare, Medicaid)	**0.77 (0.68, 0.87)**	**0.63 (0.53, 0.73)**	0.69 (0.62, 0.76)
HMO with Private Insurance	0.60 (0.54, 0.67)	**0.63 (0.57, 0.69)**	0.62 (0.57, 0.66)
No HMO, Public Insurance (Medicare, Medicaid)	0.57 (0.51, 0.63)	**0.66 (0.60, 0.72)**	0.62 (0.57, 0.66)
No HMO, Private Insurance (*referent*)	0.56 (0.50, 0.62)	0.76 (0.71, 0.80)	0.67 (0.63, 0.71)
Generalist Involved in Care			
Generalist (*referent*)	**0.61 (0.57, 0.65)**	0.69 (0.66, 0.72)	**0.66 (0.63, 0.68)**
No Generalist	0.50 (0.43, 0.58)	0.64 (0.57, 0.71)	0.58 (0.52, 0.64)
Rheumatologist Involved in Care			
Rheumatologist (*referent*)	**0.61 (0.57, 0.64)**	0.69 (0.66, 0.72)	0.65 (0.63, 0.67)
No Rheumatologist	0.47 (0.38, 0.55)	0.67 (0.61, 0.74)	0.59 (0.53, 0.65)
Total Outpatient Physician Visits*			
Fewer visits (mean - 1 SD) (5.5)	**0.54 (0.48, 0.59)**	**0.61 (0.56, 0.66)**	**0.58 (0.54, 0.62)**
Mean visits (15.8)	**0.58 (0.55, 0.62)**	**0.69 (0.66, 0.71)**	**0.64 (0.62, 0.66)**
More visits (mean + 1 SD) (26.2)	**0.63 (0.58, 0.68)**	**0.75 (0.71, 0.80)**	**0.70 (0.66, 0.73)**
***Health Status***			
SLAQ Score*			
Score at mean - 1 SD (4.7)	**0.65 (0.60, 0.70)**	0.67 (0.62, 0.72)	0.66 (0.62, 0.70)
Score at mean (12.6)	**0.59 (0.56, 0.63)**	0.68 (0.65, 0.71)	0.64 (0.62, 0.66)
Score at mean + 1 SD (20.5)	**0.53 (0.48, 0.58)**	0.70 (0.66, 0.74)	0.62 (0.59, 0.66)
SF-36 Physical Function*			
Score at mean (58.6)	**0.58 (0.55, 0.62)**	**0.69 (0.66, 0.71)**	0.69 (0.66, 0.71)
Score at mean + 1 SD (88.0)			
***Contextual Factor***	**0.49 (0.43, 0.55)**	**0.74 (0.70, 0.78)**	0.74 (0.70, 0.78)
Neighborhood Poverty			
No (*referent*)	0.59 (0.55, 0.62)	0.68 (0.65, 0.71)	0.64 (0.61, 0.66)
Yes	0.63 (0.53, 0.73)	0.74 (0.65, 0.83)	0.69 (0.63, 0.76)

Values represent adjusted pass frequencies from multivariate logistic regression models, which provide estimates of the probability of individuals receiving services after controlling for the other variables listed. Bolded values indicate *P *< 0.05. Abbreviations: SLAQ, Systemic Lupus Activity Questionnaire; SF-36, Short-Form 36 Health Survey.

*These were continuous variables in the regression models; pass frequencies represent the values at the mean score for the variable, and one standard deviation above and below the mean.

As demonstrated in the last column, the frequency of receiving preventive services increased with age but no differences were noticed between men and women, by income, or by race/ethnicity. The frequency of receiving preventive services also increased with higher educational attainment. Having a generalist physician involved in clinical care increased the likelihood of receiving preventive services, as did the total number of outpatient physician visits. Having a rheumatologist involved in care increased the likelihood of receiving immunizations. Those with managed care and public insurance have higher immunization frequencies but lower frequencies of cancer screening. Cancer screening was more likely for those with private insurance. Lastly, increased SLAQ scores and higher SF-36 physical function scores were associated with lower immunization frequencies. We did not observe a difference in the receipt of preventive services by neighborhood poverty level.

## Discussion

Although the overall receipt of cancer screening procedures and immunizations in the UCSF LOS was relatively high and comparable to the general population, we observed considerable variation among sociodemographic subgroups. In particular, individuals with SLE who were younger and reported less educational attainment were significantly less likely to receive preventive services. In addition, health system factors, such as the involvement of a generalist provider in care and total physician visits, were also associated with the receipt of preventive health care services in SLE.

Although immunization frequencies in SLE have not previously been reported, our finding that about 60% of eligible patients receive pneumococcal or influenza immunization is consistent with studies in other rheumatic diseases [22]. The finding that 40% of patients remain unvaccinated is notable given that approximately one-third of deaths in SLE are attributable to infections [23], and several studies have found that respiratory infections, including those attributable to *Streptococcus pneumoniae*, are the leading cause of serious infections in SLE [24,25]. We did not investigate specific reasons for non-immunization, which might include patient preferences, contraindications, failure of providers to offer the vaccine, or cost. In SLE, suboptimal immunization frequencies may also partly reflect long-standing concerns about vaccine safety that arose after early reports of fatalities or increased disease activity following immunization [26,27]. However, several decades of scientific research have now failed to corroborate these early concerns [28]. Based on this evidence, recently developed quality indicators for SLE have established influenza and pneumococcal immunization in this population as a minimally acceptable standard of care [13]. Our study suggests that individuals with lower educational attainment are at particularly high risk of not receiving immunizations, a finding that is consistent with a growing body of evidence that individuals with less education may receive poorer quality health care [29,30].

About one third of patients in our cohort did not receive recommended cancer screening tests. As in the case with immunizations, younger patients and those with less educational attainment were less likely to receive recommended cancer screening. Although we expected that those enrolled in HMOs would be more likely to receive cancer screening given the emphasis of these organizations on preventive care, this was not the case. However, consistent with other recent studies, individuals with a higher number of physician visits were more likely to receive cancer screening services [31,32]. No specific cancer surveillance guidelines for SLE have been published to date, although at a minimum, it seems reasonable that SLE patients should receive cancer surveillance tests recommended for the general population. One previous study has examined cancer surveillance rates in Canadian SLE patients using a tertiary-care clinic cohort. In that study, researchers surveyed 146 patients regarding receipt of cancer screening tests in the last year. Unlike our study that found screening rates similar to the general population, rates in that cohort fell well below general population cancer screening rates in Canada [5].

Our study also suggests that both a higher number of physician visits and involvement of primary care providers in SLE management are associated with improved preventive health care quality. As short-term mortality from SLE declines and clinical care increasingly shifts toward the long-term prevention and management of co-morbidities and complications, effectively coordinating care between specialists and primary care providers is a priority. Such coordination, however, may prove challenging in SLE, where clinical care often spans multiple specialists who sometimes practice at significant geographic distances from each other [33,34]. Increasing awareness among primary care providers about the higher risk of specific long-term complications in SLE, and improving care coordination by specialists caring for SLE patients through various practice innovations, such as automatic reminders in electronic medical records, may help improve the quality of preventive health care in this population.

Studying health services in SLE is challenging given the low prevalence of the disease. Most research to date has drawn from tertiary care specialty clinics, which may not adequately represent the entire population with SLE. Although not a random sample of the population of patients with SLE, our study has attempted to partly address this gap by drawing from community-based sources. Nevertheless, our study has limitations. First, because the LOS participants speak English and have health insurance, we cannot generalize our findings to non-English speaking individuals or those without health insurance - two groups that are at great risk of receiving poorer quality health care. Conducting this research in non-English speaking and indigent populations should be prioritized in future studies. Second, participants in the LOS may not be directly comparable to those in CHIS. Although we had a sufficient number of observations to standardize our sample based on age and education and included only insured individuals in our analyses, additional standardization by potential confounding factors such as race/ethnicity and type of health insurance was not possible. Third, our study did not include at least one variable found in previous research to influence the receipt of preventive services: medical comorbidities [31]. Incorporating a measure of comorbidity in future studies on the topic is therefore warranted. Fourth, our study did not assess the specific reasons for not receiving preventive services, and therefore we cannot determine whether gaps in care are secondary to patient, provider or health system factors. Finally, our data derive from self-report. Previous studies have demonstrated that self-report is a reasonable, although not perfect, proxy for various preventive health services [31,35-38].

## Conclusions

Our findings demonstrate that there remains room for improvement in the quality of care for two important categories of preventive service in SLE, particularly in younger patients and those with lower educational attainment. Although individuals in the LOS appear to receive preventive services at frequencies similar to CHIS, receipt of services in both groups is suboptimal. Given the higher burden of infections and malignancies in SLE, identifying strategies to abate these long-term outcomes in SLE is an important goal. Coordinated, multidisciplinary care that involves generalist providers may offer an opportunity to improve the provision of preventive health services in SLE.

## Abbreviations

CDC: Center for Disease Control; CHIS: California Health Interview Survey; HMO: health maintenance organization; LOS: Lupus Outcomes Study; SF-36: Short-Form 36; SLAQ: Systemic Lupus Activity Questionnaire; SLE: systemic lupus erythematosus; UCSF: University of California San Francisco.

## Competing interests

The authors declare that they have no competing interests.

## Authors' contributions

JY, PP, AH, LJ, PK and EY contributed to the study design. JY, LT, PK, LC and EY contributed to acquisition of data. JY, CT and EY contributed to analysis and interpretation of data. JY, PP, JZG, AH, PK, LC and EY contributed to manuscript preparation. JY, CT and LT contributed to statistical analysis.
